# A Simple Whole-Plasmid PCR Method to Construct High-Diversity Synthetic Phage Display Libraries

**DOI:** 10.1007/s12033-021-00442-4

**Published:** 2022-02-02

**Authors:** Maria T. Tsoumpeli, Alison Gray, Aimee L. Parsons, Anastasios Spiliotopoulos, Jonathan P. Owen, Keith Bishop, Ben C. Maddison, Kevin C. Gough

**Affiliations:** 1 School of Veterinary Medicine and Science, The University of Nottingham, College Rd., Sutton Bonington, Leicestershire LE12 5RD UK; 2 Vertex Pharmaceuticals (Europe), 88 Jubilee Avenue, Milton Park, Abingdon, Oxfordshire OX14 4RW UK; 3 ADAS Biotechnology, School of Veterinary Medicine and Science, The University of Nottingham, College Rd., Sutton Bonington, Leicestershire LE12 5RD UK

**Keywords:** Phage display, VHH, Nanobody, Whole-plasmid PCR, Antibody, Peptide, Phagemid, Phage library

## Abstract

Phage display technology utilises peptide and antibody libraries with very high diversities to select ligands with specific binding properties. The production of such libraries can be labour intensive and technically challenging and whilst there are commercial sources of libraries, the exploitation of the resulting binders is constrained by ownership of the libraries. Here, a peptide library of ~ 1 × 10^9^ variants for display on gene VIII was produced alongside three VHH antibody libraries with similar diversity, where 12mer, 16mer or 21mer CDR3s were introduced into the highly stable cAbBCII10 scaffold displayed on gene III. The cloning strategy used a simple whole-plasmid PCR method and type IIS restriction enzyme assembly that facilitate the seamless insertion of diversity into any suitable phage coat protein or antibody scaffold. This method reproducibly produced 1 × 10^9^ variants from just 10 transformations and the four libraries had relatively low bias with 82 to 86% of all sequences present as single copies. The functionality of both peptide and antibody libraries were demonstrated by selection of ligands with specific binding properties by biopanning. The peptide library was used to epitope map a monoclonal antibody. The VHH libraries were pooled and used to select an antibody to recombinant human collagen type 1.

**Supplementary Information:**

The online version contains supplementary material available at 10.1007/s12033-021-00442-4.

## Introduction

Phage display technology was first described in 1985 by George Smith [1] and has been developed to include the display of peptides, various recombinant antibody formats, enzymes and fragmented proteomes [2–4]. These phage libraries can display vast diversities of ligands on coat proteins projecting from the surface of the bacteriophage particle, utilising either a phagemid or phage vector system to encode the ligand-coat protein fusion [5–7]. In general, relatively small ligands such as peptides are usually displayed on either coat protein pIII or pVIII and larger protein fragments such as those derived from antibodies are usually displayed on pIII. Phage display libraries can enable the identification of a variety of ligands with desirable binding properties including ligands that bind to toxic or non-immunogenic antigens.

Whilst there are commercial sources of both peptide [8] and antibody [9] libraries, the exploitation of any isolated ligand is often constrained by ownership of the libraries. It may therefore be required to produce bespoke synthetic one-pot ligand libraries containing a high diversity of potential binders that can provide antibodies or peptides to any target antigen with no constraints on use.

Synthetic peptide libraries are usually cloned by the annealing of overlapping complementary oligonucleotides, one of which codes for the peptide diversity, followed by complementary strand synthesis and digestion of the products with restriction enzymes to facilitate cloning into the phage or phagemid vector [10]. Alternatively, short oligonucleotides can be annealed to either side of the region of diversity encoded within a longer oligonucleotide such that they form sticky ends compatible with direct ligation into restriction enzyme cut vector [11]. The circularised vector, containing a short single-stranded gap, is then used for bacterial transformation. Recently, Kong et al*.* [12] have described a methodology for the production of high-diversity peptide libraries using a whole-plasmid PCR self-ligation process which vastly simplifies and increases the efficiency of library construction.

In terms of antibody formats that are amenable to phage display, Fabs consist of the VH–CH1 and VL–CL chains, one of which is fused to the pIII protein [13]. ScFvs consist of just the VH and VL chains joined together by a peptide linker with one chain fused to the pIII protein [14], and VHHs consist of a single heavy chain domain equivalent to a conventional IgG VH domain [15]. VHHs, also known as single-domain antibodies or nanobodies, are derived from natural heavy chain antibodies found in camelids that lack a light chain. Synthetic antibody libraries are usually based on a single or very low number of “scaffold” sequences with diversity introduced into one or more of the complementarity determining regions (CDRs). Conventional cloning of Fab and scFv libraries use multiple PCRs to introduce diversity into CDR regions and then an overlapping extension PCR to amplify the scFv or separate VL and VH chains for Fabs. The products are then cloned using multiple restriction enzymes compatible with the phage or phagemid vector (for example [16, 17]). A variation on this strategy is the use of type IIS restriction enzymes to facilitate the so-called ‘seamless’ cloning of CDR regions [18]. As this type of restriction enzyme cuts outside its recognition sequence, an overhang compatible with any sequence in the scaffold can be produced. Subsequent cloning results in a DNA sequence devoid of any restriction enzyme site and therefore addition of extra amino acids within expressed sequences can be avoided and the process is irreversible. Alternative methods use a variation on Kunkel mutagenesis to introduce diversity into multiple CDRs simultaneously by annealing oligonucleotides (containing diversity) to CDR regions in scFv–phagemid template ssDNA and then synthesising the second strand of the DNA to produce heteroduplex DNA that is used directly for transformation [19] or is further amplified by rolling circle amplification before transformation [20].

There are several commercially available Fab libraries, for example, the Ylanthia library from MorphoSys [21]. This library has very high diversity and is based on 36 fixed heavy/light chain pairs selected from 400 combinations that were characterised for desirable biophysical properties such as high expression levels and high thermal stability. CDRH1 and CDRH2 had limited diversity introduced and high diversity was then introduced into CDRH3 and CDRL3, to produce a library of ~ 1 × 10^11^ clones. The authors used trimer-phosphoramidite-oligonucleotides or a proprietary gene synthesis technology called Slonomics® to produce oligonucleotides that tightly control the codons inserted in the regions of diversity [22]. The synthetic diversified gene regions were then cloned into the scaffold using type IIS restriction enzyme cloning.

Examples of scFv libraries include the Tomlinson I and J library [9] that was produced by mutating CDR2 and CDR3 regions of both VL and VH in a single scFv scaffold [23], or the HuCal library that introduced diversity into CDR3s [24, 25]. The HuCal library was produced using 7 VL and 7 VH scaffolds to produce 49 combinations into which CDR3 diversity was introduced using trimer-phosphoramidite-oligonucleotides [24] to yield a library with diversity of ~ 2 × 10^9^. Cloning of the CDR diversity was carried out using engineered restriction sites within the scaffolds flanking the CDR3 domains. An alternative approach to scFv library construction used the modular cloning of different CDR2 and CDR3 regions [20]. Antibody sequence databases were mined for heavy and light chain CDR2 and CDR3 sequences contained within a particular scFv framework. Based on 2000 sequences, shuffling of the 4 CDR regions produced a library of ~ 1 × 10^10^ diversity. The library assembly used Kunkel mutagenesis to introduce the distinct CDR regions into the scaffold clone followed by Rolling Circle Amplification to further amplify products before ligation and transformation.

A single-pot, synthetic VHH phage display library has also been reported. This used the cAbBCII10 VHH template as a scaffold for grafting in CDR3 diversity [15]. This scaffold has high stability and expression levels, lacks the CDR1–CDR3 disulphide bond and can be expressed in the cytoplasm. A ~ 1 × 10^9^ diversity library consisting of random 16mer CDR3 inserts was produced. The library was cloned using an overlapping PCR assembly method with the overlap in the FR3 domain to amplify the full VHH gene followed by digestion with multiple restriction enzymes to facilitate cloning into a phagemid vector.

Here, we describe a simple single-step whole-plasmid PCR methodology for re-creating the phagemid backbone together with a region of library diversity, similar to a procedure recently described [12]. Our methodology builds on coupling the library DNA synthesis step with a type IIS restriction enzyme assembly to introduce high levels of diversity into phagemid vectors for the production of novel display libraries. This process allows seamless cloning of random sequence to be achieved, without the introduction of restriction enzyme sequence that would add additional amino acids to the expressed product. In doing so, the method can be used to create antibody libraries where diversity can be introduced to a specific site, such as specific CDR domains within the antibody scaffold. Here, the method was used to create a peptide library of 5 × 10^9^ variants for display on protein VIII and also to produce a VHH library with similar diversity where 12mer, 16mer or 21mer CDR3 random sequences were introduced into the highly stable cAbBCII10 scaffold for display on protein III. Molecular analysis of the resulting libraries (sequence verification and determination of library diversity by NGS) as well as functional testing (isolation of ligands to target antigens by biopanning) were carried out in order to validate the libraries.

## Methods

### Library Construction

**Fig. 1:**
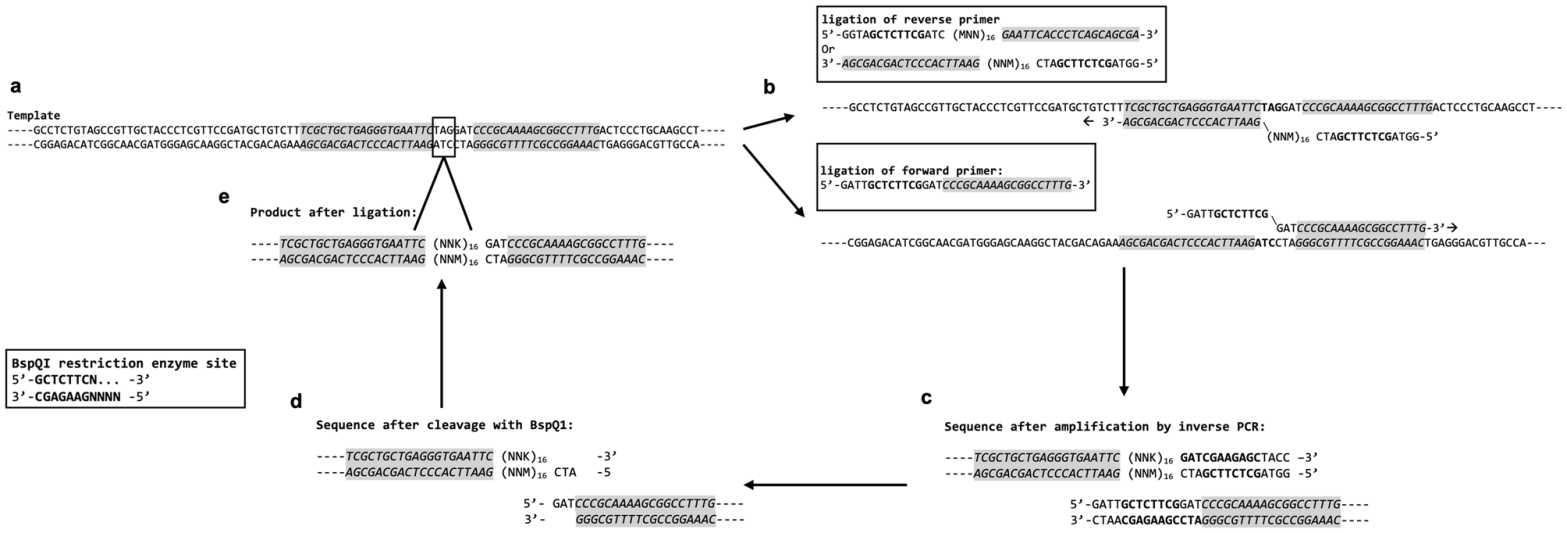
The construction of high-diversity ligand libraries by whole-plasmid PCR. The example shown is for the pC89 pVIII-16mer peptide library where the phagemid vector has a TAG stop codon within the insertion site that is removed during library cloning (boxed, **a**). Primers are designed containing type IIS restriction enzyme cleavage sites (in this case BspQI/SapI, in bold) and contain 20 bp regions homologous with the phagemid sequence either side of the insertion site (shaded and italics, **b**). Restriction enzyme sites are formed after whole-plasmid PCR (**c**) and compatible sticky ends produced after cleavage (**d**). Ligation (**e**) results in seamless cloning where the TAG codon is replaced by the 16mer peptide sequence with no cloning-derived residues inserted. An analogous strategy allows the insertion of areas of diversity within any cloned scaffold, for example, the CDR region(s) of antibodies

A 16mer peptide library was cloned as a fusion to the pVIII phage coat protein gene sequence (designated pC89 pVIII-16mer) using the phagemid vector pC89 [26] (kindly provided by Professor Franco Felici, University of Molise, Italy). A BspQI/SapI site was removed from pC89 by a site-directed mutagenesis by whole-plasmid PCR using phosphorylated reverse primer A, 5′-TTCCTCGCTCACTGACTC-3′ and phosphorylated forward primer B, 5′-GCGGAGGACGCCCAAT-3′. PCR products were resolved by agarose gel electrophoresis and a 3.4 Kb band was excised and purified (Nucleospin, Macherey Nagel). DNA was blunt end ligated and transformed into TG1 electrocompetent *E. coli* (Agilent). Transformants were sequenced to confirm the removal of the BspQI/SapI cleavage site. The modified pC89 vector was then used as a whole-plasmid PCR template to introduce random 48 bp DNA inserts fused to the coding sequence for the phagemid encoded gene VIII. Peptide diversity was introduced within the reverse primer where PCR Forward primer 5′-GATTGCTCTTCGGATCCCGCAAAAGCGGCCTTTG-3′ and the reverse primer 5′-GGTAGCTCTTCGATC(MNNx16)GAATTCACCCTCAGCAGCGA-3′ were used. Whole-plasmid PCR, which reconstituted the complete PC89 vector as a single linear PCR product was carried out using Q5 polymerase (NEB), annealing temperature 57.5 °C with 2 min 20 s elongation at 72 °C per cycle (see Fig. 1 for details). PCR fragments of 3.4 kb were gel purified and pooled, 1 μg of this DNA was then digested with the type IIS restriction enzyme SapI and then DpnI to remove template DNA. Digested DNA was column purified (Nucleospin kit, Macherey Nagel) according to the manufacturer’s instructions. 1–2 μg of pooled DNA was then ligated with 2 μl of T4 DNA ligase (4000 U; NEB) and column purified (Nucleospin kit, Macherey Nagel) and eluted in water. 1 μg aliquots of ligated product were transformed into 50 μl of TG1 electrocompetent cells (Lucigen) using 1-mm gap width cuvettes (Bio-Rad) and a 2.5 kV pulse, and 17 transformations carried out. Transformants were selected on LB-Agar supplemented with ampicillin. Ampicillin-resistant transformants were titred to estimate library diversity. A number of transformants were isolated and Sanger sequenced using primer 5′-CTTTATGCTTCCGGCTCGTATG-3′ to confirm the presence of DNA fragments coding for 16mer peptide.

Three VHH libraries were synthesised for display of antibody on pIII using the phagemid vector pSD3 [27]. The vector was digested with SfiI and SpeI to remove the region between the pelB leader sequence and the truncated pIII gene. A gene sequence for the VHH CAbBII10 scaffold [28] with a C-terminal fusion to the Strep II tag [29], the residues Ala-Gly-Ala and a 6xHis tag followed by an amber stop codon flanked by an upstream SfiI cleavage site and a downstream SpeI site was synthesised with *E. coli* optimised codon usage (Biomatik) and supplied cloned and sequenced within the vector pUC57. The Sfi–SpeI fragment from this construct was subcloned into the pSD3 vector (Fig. S1). As with pC89 this pSD3-VHH construct also contained a BspQI/SapI cleavage site within the vector backbone. This was removed by whole-plasmid PCR using the 5′ phosphorylated primers, reverse 5′-AGCTACGCGCTTCCTCGCTCAC-3′ and forward 5′-CGTAGCTGCCCAATACGCAAAC-3′. The resulting pSD3-VHH construct was used as the template to produce libraries of random peptides of 12, 16 and 21 amino acids in length inserted within the CDR3 site. The forward primer was the same for all 3 VHH libraries: 5′-GCGCTCTTCTTGGGGACAAGGGACGCAG-3′. The reverse primer differed in the randomised region size: 5′-GTGCTCTTCCCCA(MNNx21)CGCAGCGCAATAGTAGATCGCG-3′, 5′-GTGCTCTTCCCCA(MNNx16)CGCAGCGCAATAGTAGATCGCG-3′ and 5′-GTGCTCTTCCCCA(MNNx12)CGCAGCGCAATAGTAGATCGCG-3′ for the 21, 16 and 12mer peptide libraries, respectively. The library construction steps were as described above with the exceptions that the PCR annealing temperature was a gradient between 50 and 65 °C, type IIS BspQI was used instead of SapI and 10 transformations were carried out for each library with between 0.7 and 1.1 μg of DNA.

A limited number of individual clones were picked, phagemid DNA was miniprepped (Qiagen) and the region of diversity was analysed by Sanger sequencing using the primer 5′-TATTTCAAGGAGACA-3′.

### NGS analysis of libraries

To further assess diversity and bias within each library, NGS analysis was carried out. DNA from the pC89 peptide library was purified using a Miniprep Kit (Qiagen) from glycerol stocks of the library, and 10 ng was amplified with primers p8_forward: 5′-GTAATCCTTGTGGTAGTATCGGATGCTGTCTTTCGCTGC-3′ and p8_reverse: 5′-CTAGAACATTTCACTTACGGTTTTCCCAGTCACG-3′ using high-fidelity Q5 polymerase. PCR was carried out with an initial incubation for 3 min at 95 °C, followed by 30 cycles of denaturation for 30 s at 95 °C, annealing for 30 s at 60 °C and extension for 30 s at 68 °C, and a final single incubation for 5 min at 72 °C. DNA was purified using the Nucleospin kit (Macherey Nagel) according to the manufacturer’s instructions. 10 ng of DNA was then amplified in a second PCR using primers p1: 5′-CCTCTCTATGGGCAGTCGGTGATCTAGAACATTTCACTTAC-3′, and barcoded primer, 5′-CCATCTCATCCCTGCGTGTCTCCGACTCAG*TCCGACAAGC*GTAATCCTTGTGGTATCG-3′ (the barcode sequence in italics varied for each sample) as for the previous PCR but for 12 cycles using an annealing temperature of 63 °C. This second PCR attached both a new adapter sequence for compatibility with the Ion Proton sequencing platform and a unique barcode sequence, for identification and analysis. The sample was resolved on a 3% (w/v) Metaphor agarose gel and the DNA band size of 330 bp was extracted and purified. A second purification step used the Agencourt AMPure XP Bead Clean-up kit (Beckman Coulter) according to the manufacturer’s instructions. The concentration of DNA was measured with a Qubit dsDNA HS Assay Kit (ThermoFisher) using Qubit 2.0 Fluorometer 2.0 (Invitrogen).

The VHH libraries were prepared using the same method but using the primers 5′-GTAATCCTTGTGGTATCGCTAGAGGAAACTGTCACCTG-3′ and 5′-CTAGAACATTTCACTTAGATTCGGTAAAGGGCCGC-3′ to amplify the peptide diversity region. This amplicon was then re-amplified in the second PCR as above but using a different unique barcode sequence for each library. Barcoded DNA samples for all 4 libraries were pooled and sequenced on the Ion Proton platform with an Ion PI Chip (University of Pennsylvania, US).

Perl scripts were used to process NGS data files as previously described [30]. Briefly, FASTQ files were converted to FASTA files and Ion Torrent barcodes identified using the “FASTQ/A Barcode splitter” (part of the FASTX-toolkit from http://han-nonlab.cshl.edu/fastx_toolkit/index.html). DNA sequences in each barcode-binned FASTA file were translated in all 3 frames and concatenated to a single file (translate.pl). FASTA files were processed to identify matching flanking sequence motifs (AEGEF and DPAKAA for the pC89 peptide library and YYCAA and WGQGT for the VHH libraries) to capture insert (peptide/HCDR3) sequence. Parameter choices for data analysis were as follows: maximum 2 mismatches allowed per barcode; minimum of 1 amino acid peptide between flanking motifs for analysis; and amber stop codon (TAG) replaced with amino acid Q.

### Panning

The peptide phage display library was grown to mid-log phase (OD_600_~0.6), superinfected with M13KO7 helper phage (Pharmacia LKB) and incubated overnight at 30 °C (220 rpm) to produce phage particles. Phages were harvested and PEG precipitated and biopanning was carried out as previously described [31]. Briefly, anti-prion protein (PrP) antibody SAF84 (1/2000 in 0.5 ml PBS, obtained from J. Grassi, CEA Saclay, Gif/Yvette, France) was coupled to Pierce™ Protein G Agarose beads [50 μl of a 50%(w/v) suspension], with rotation for 1 h at room temperature (RT). Beads were washed 3 times with PBS, blocked with 300 μl of PBS with 3%(w/v) non-fat dry milk (MPBS) for 1 h at RT and washed a further three times with PBS. Meanwhile, to block non-specific interactions, 1 ml of the phage was pre-incubated with blocked, uncoupled protein G beads in MPBS for 1 h before removal by centrifugation at 2000×*g* for 3 min. To isolate specific binders, the phages were then incubated with the anti-SAF84-beads for 3 h, with rotation at RT, followed by 5 washes with PBS with 1%(v/v) Tween (PBST) and 5 washes with PBS. Glycine HCl (100 μl of 0.2 M, pH 2.6) was used to resuspend the pelleted beads and the sample incubated at RT for 10 min. Beads were pelleted, eluted phages were removed and neutralised by the addition of 100 μl 1 M Tris buffer pH 7.4. Neutralised phage (100 μl) was added to 10 ml of mid-log TG1 bacterial culture. Bacteria were plated out with ampicillin selection.

The 3 VHH libraries were prepared by pooling equal numbers of TG1 from glycerol stocks of each of the libraries (ratio 1:2:3 by volume for libraries pSD pIII-VHH_21mer, pSD pIII-VHH_12mer and pSD pIII-VHH_16mer, respectively). Subsequent steps were as described above [31], except that bacteria were superinfected with Ex-12 helper phage [32] prior to each of three iterative rounds of biopanning (using ~ 10^12^ phage/ml, 1 ml). Each round of phage selection was carried out against recombinant human collagen type 1 (Merck) prepared at 20 μg/ml in fibrillogenesis buffer (16.2 mM sodium phosphate dibasic pH11.2) and pre-incubated at 100 μl/well on Nunc maxisorb plates (ThermoFisher) for 16 h at 24 °C. Phages were incubated with antigen for 1.5 h at RT, followed by 10 washes with PBST and 10 washes with PBS. After 3 rounds of biopanning, monoclonal VHHs were obtained by picking individual ampicillin-resistant TG1 colonies.

### ELISAs

Phage ELISA: cultures of polyclonal phage or single phage were used to produce peptide phage or VHH phage particles by rescue with M13KO7 or Ex-12 helper phage, respectively, as described above for library phage but without a phage supernatant precipitation step. Target proteins (SAF84 at 2 μg/ml or human collagen type 1 at 20 μg/ml) were coated onto Nunc MaxiSorp plates (ThermoFisher) overnight at 4 °C or 24 °C, respectively (100 μl per well). Control antigens were also used: anti-PrP antibody SAF70 (2 μg/ml) for SAF84 binders and elastin (20 μg/ml in carbonate buffer) for collagen binders. The plates were then washed 3 times with PBS and each well blocked with 400 μl of MPBS for 1 h at RT. After washing 3 times with PBS, 100 μl of phage supernatant (pre-incubated with MPBS for 1 h) was added per well and incubated for 1 h, followed by 3–5 washes with PBST and 3 with PBS. For the peptide phage,100 μl of rabbit anti-fd bacteriophage antibody (Sigma, 1/2000 in MPBS) was then added followed by 100 μl of mouse anti rabbit-alkaline phosphatase conjugate (Invitrogen, 1/2000 in MPBS). For the VHH phage, binding was detected with mouse anti-M13 bacteriophage monoclonal antibody (E1) biotin (Invitrogen, 1/1000), followed by streptavidin–alkaline phosphatase conjugate (Sigma, 1/1000). Alkaline phosphatase substrate p-Nitrophenyl phosphate substrate buffer (Sigma) was added (100 μl per well). Plates were read at 405 nm after colour development.

The MBP–VHH ELISA was carried out as described above to detect 10 μg/ml human collagen type 1 to V and recombinant collagen type I (all Sigma, immobilised in PBS) with pure MBP–VHH (used at 12.7 μg/ml, 100 μl per well) and binding was detected with mouse anti-MBP antibody (New England Biolabs (NEB), 1/10,000) and rabbit anti-mouse IgG alkaline phosphatase conjugate (Invitrogen, 1/1000). Binding to a control antigen (tropoelastin, at 10 μg/ml) was also carried out.

### Subcloning and Expression of a VHH Clone

The VHH CAbBII10 gene was cloned into pMal-c5x using the EcoRI and NcoI restriction sites. The anti-collagen CDR3 region was then cloned by whole-plasmid PCR into this c5x-VHH using the following primers: phosphorylated forward 5′-CCGTCTCAGCGTGACCTGTGGGGACAAGGGACGCAG-3′ and reverse 5′-AGCACCCCAAGCGGTGATCGCAGCGCAATAGTAGAT-3′. Reactions used Q5 polymerase and PCR conditions described for the VHH library construction. Whole-plasmid PCR product was self-ligated and transformed into electrocompetent *E. coli* TG1 (Agilent).

Vector pMal-c5x containing the gene for the anti-collagen MBP–VHH fusion was then transformed into *E. coli* ER2523 cells. For soluble VHH expression, cells were grown to mid-log in a 1 L culture of 2TY supplemented with ampicillin and 0.2% (w/v) glucose, at 37 °C, with shaking at 220 rpm. Expression was induced at mid-log by replacing the media with fresh 2TY containing 0.1 mM isopropyl b-D-thiogalactopyranoside (IPTG) and the cells grown for 3 h at 30 °C. Cells were harvested by centrifugation for 10 min at 3500×*g*. Cells were lysed by re-suspension in BugBuster Protein Extraction Solution (Merck) containing 300 μg/ml lysozyme, 25 U/ml benzonase and 1 × complete protease inhibitor cocktail (Roche) and incubation for 1 h at RT with rotation. The cell lysate was clarified by centrifugation for 30 min at 16,000×*g*. A one-step purification of the MBP fusion protein was carried out under native conditions on an amylose resin following the manufacturer’s instructions (NEB). Flow through, wash and eluate fractions (10 μl of each) were analysed on a 12%(w/v) polyacrylamide gel (precast NuPAGE SDS-PAGE Bis–Tris; Invitrogen) and stained with InstantBlue (Expedeon).

## Results

### The Production of Whole-Plasmid PCR Libraries

Four libraries were produced, one 16mer peptide library displayed on pVIII (designated pC89 pVIII-16mer) and three VHH libraries displayed on pIII protein. The latter were based on a single scaffold (CAbBII10) with diversity introduced by the insertion of random peptides of 12, 16 and 21 amino acids in length within the CDR3 site (designated pSD pIII-VHH_12mer, pSD pIII-VHH_16mer and pSD pIII-VHH_21mer, respectively). CDR3 was randomised as this is the most diverse region of natural VHHs with the greatest contribution to epitope binding [33]. All libraries used the same BspQI/SapI seamless cloning strategy (example given in Fig. 1) with diversity introduced using NNK codons (present in the reverse primer as 5′-MNN-3′). For pC89 pVIII-16mer, 17 transformations resulted in an estimated 5 × 10^9^ transformants. For the VHH libraries containing 12, 16 or 21mer inserts, 10 transformations for each yielded 2 × 10^9^, 3 × 10^9^ and 1 × 10^9^ transformants, respectively.

### Analysis of Library Diversity and Bias

Sanger sequencing of 20 clones after peptide-pVIII library construction (data not shown) indicated that 80% had an in-frame 16mer insert, 10% had a shorter in-frame insert and 10% had no insert. For the VHH libraries, Sanger sequencing of 18 clones from the 12mer and 16mer libraries demonstrated that 94% contained the VHH CDR3 insert of the correct size and in frame, for the 21mer library 67% (of 18 clones) were of the correct size and in frame. Other clones had out of frame inserts, most likely due to primer errors being more frequent in the longer primer required to introduce a 21 amino acid region of diversity.

**Fig. 2:**
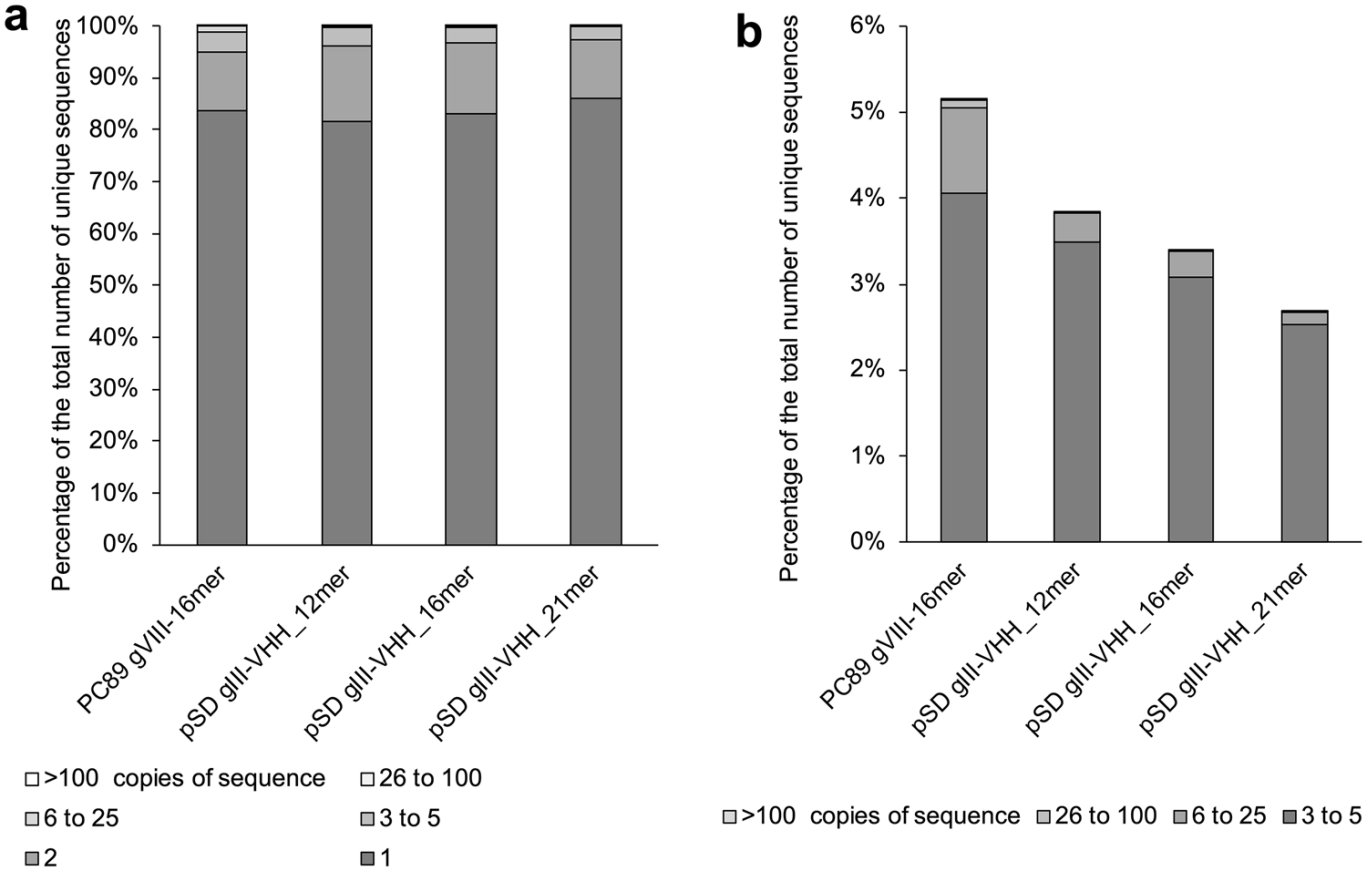
Insert sequence diversity of ligand libraries. Libraries contained a similar level of singleton clones (82 to 86%, **a**) and only ~ 3–5% of sequences were present at a copy number of greater than 3 (**b**)

NGS analysis was carried out for both libraries. In-frame sequences producing intact flanking regions (AEGEF and DPAKA for pC89 pVIII-16mer and YYCAA and WGQGT for the VHH libraries) were analysed and revealed that 73 to 85% of the clones were distinct peptides of which 82 to 86% were present as single copies; between 531,000 and 928,000 sequences were analysed for each library (Table 1, Fig. 2). The correct size insert was present in 90 to 98% of the clones. In theory, the NNK codon does not introduce any ochre or opal stops but NGS analyses identified that 0.026% of codons were these stop codons and will result in non-functional clones (which for 12mer to 21mer inserts represent ~ 0.9 to 1.6% of all clones), it is not known whether these non-NNK codons are due to sequencing errors [34]. As with other libraries [34, 35], bias was present in the randomised sequences. Calculating Poisson probabilities (taking into account the depth of sequencing and diversity of each library to calculate the expected value), the probability of seeing a sequence with a copy number of 1 was between 0.977 and 0.999, being seen twice was between 9 × 10^–5^ and 1 × 10^–4^ and being seen three times between 6 × 10^–9^ and 1 × 10^–7^ (Table S1). At the sequencing depth performed (5 to 9 × 10^5^), it should have been a very rare event to see any sequence greater than 3 times, yet sequences with a copy number of 3 or more made up between 3.4 and 5.3% of the sequences seen. Bias was greater in the pVIII-displayed peptide library compared to the pIII-displayed VHH libraries. The most overrepresented peptides, present at greater than 6 copies, represented 0.98% of the pVIII library but just 0.14 to 0.33% of the VHH-pIII libraries (Fig. 2). The VHH libraries did not contain any sequence above 0.006% of the sequences (33 copies, Table S2) with the exception of the original CAbBCII10 template sequence VRGYFMRLPSSHNFRY that was present in between 0.02 and 0.03% of sequences. For the peptide library, the most overrepresented sequences were those containing DCSS, a sequence from codons present in the uncut forward primer. These represented 2.4% of all sequences containing the in-frame flanking regions. The library also contained clones with a TAG stop codon as insert, which was the template phagemid and represented 0.28% of sequences containing the in-frame flanking regions (Table S2). The equivalent of the DCSS sequence present due to aberrant primer does not occur for the reverse primer used to produce this library, or in the VHH libraries. This suggests that the DCSS containing inserts may be due to errors in the forward primer synthesis used in the pVIII peptide library construction. The primers used for the peptide library construction were commercially sourced at 65–80% pure, and those used for the VHH libraries at > 85% pure.

**Table 1:** Summary of NGS analysis of 4 phage display libraries

	PC89 pVIII-16mer	pSD3 pIII-VHH_12mer	pSD3 pIII-VHH_16mer	pSD3 pIII-VHH_21mer
Total sequences^a^	928,608	866,825	836,873	531,084
Distinct sequences^b^	678,851 (73%)	699,656 (81%)	686,835 (82%)	450,011 (85%)
Singletons	567,972 (84%)	570,611 (82%)	570,359 (83%)	387,623 (86%)
Repeat sequences	110,879 (16%)	129,045 (18%)	116,476 (17%)	62,388 (14%)
Correct insert^c^	611,127 (90%)	680,375 (97%)	672,374 (98%)	433,311 (96%)

^a^All sequences that contained the two flanking regions in frame with each other either side of the insert site

^b^Different peptide sequences as a percentage of the total sequences, these will be present as singletons or repeated sequences

^c^Sequences that contained an insert of the expected size

**Fig. 3:**
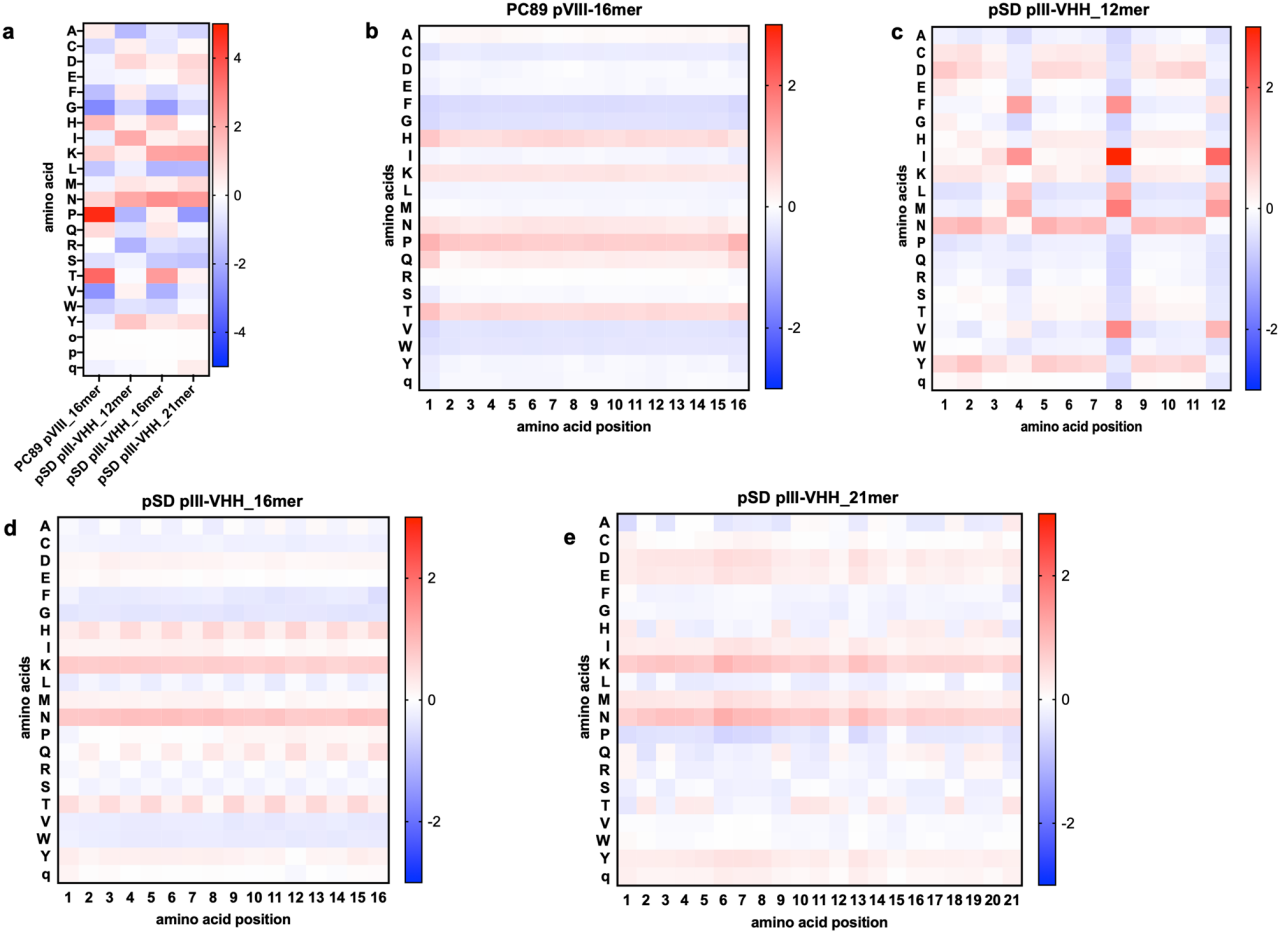
Amino acid usage in diverse ligand libraries. The over- or underrepresentation of each amino acid was calculated by comparison to the theoretical amino acid codon usage from the NNK codon. The heat map shows the percentage difference in the display frequency from the theoretical value (**a**). Similarly, bias for each amino acid was determined for each position within the areas of diversity (**b** to **e**, for the PC89 pVIII-16mer, pSD pIII-VHH_12mer, pSD pIII-VHH_16mer and pSD pIII-VHH_21mer libraries, respectively)

Considering amino acid usage in the 4 libraries, for the gene VIII peptide library the residues Pro and Thr were the most overrepresented, by 4.8 and 3.5%, respectively (Fig. 3a). Val and Gly were the most underrepresented, by − 2.6 and − 2.8%, respectively. Overall, amino acid usage bias was not as pronounced for the three pIII libraries produced within the VHH scaffold gene, all had Asn as the most overrepresented amino acid and the highest bias was just 2.6%. The most underrepresented residue was different for each library and was never greater than − 2.5% (Pro in library pSD pIII-VHH_21mer). In terms of amino acid positional bias, for the pVIII peptide library this was consistent across all residues but with some increased bias at residues 1 and 16 where Pro was more overrepresented, but no bias was greater than 1.1% of the theoretical distribution (Fig. 3b). The VHH libraries displaying 16mer and 21mer peptides had very similar patterns of amino acid positional distribution with Asn and Lys being the most overrepresented at all residues (Fig. 3d, e) and no amino acid bias was greater than 1.2%. With the pSD pIII-VHH_12mer library, the pattern of amino acid distribution was distinct from the other VHH libraries; whilst Asn was overrepresented at most residues this was not the case at residues 4, 8 and 12. At these residues Ile was overrepresented by up to 3.0% (Fig. 3c).

### Selection of Functional Clones from the Libraries

To demonstrate that the libraries with diversities of ~ 1 × 10^9^ variants in a single region could produce functional clones with specific binding properties, the peptide library was used to epitope map a monoclonal antibody and the VHH libraries were combined and used to select a binder to a target protein.

**Fig. 4:**
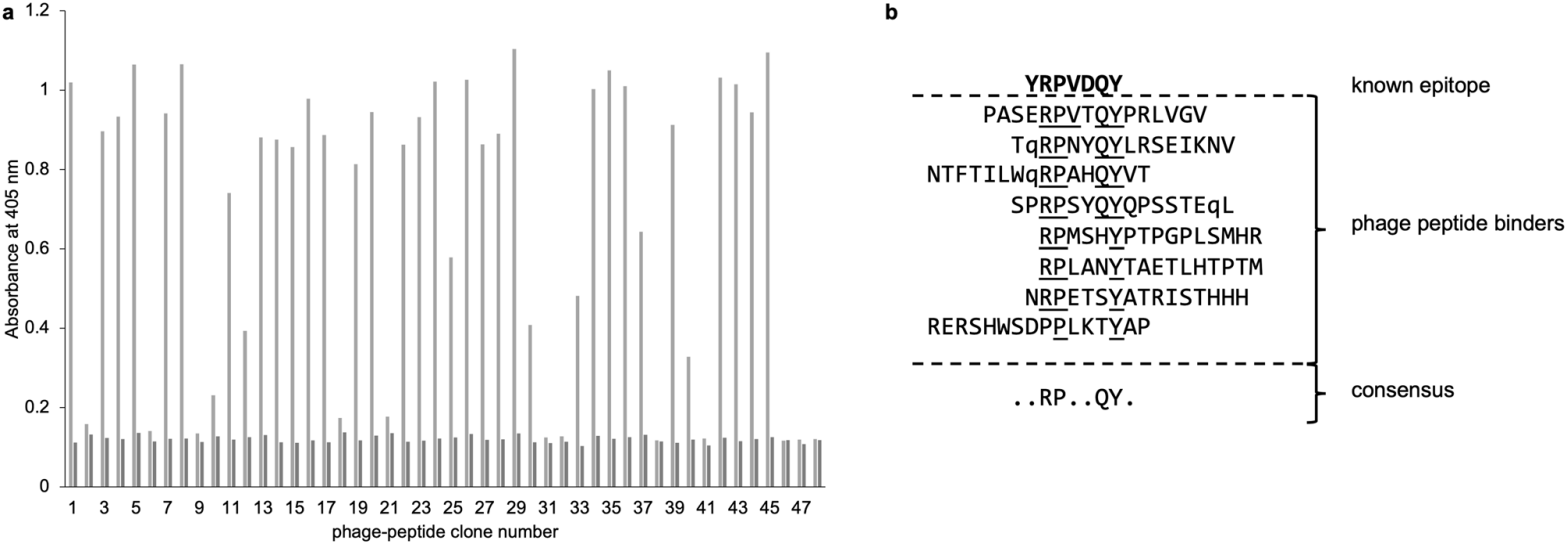
Epitope mapping using the PC89 pVIII-16mer library. A single round of panning was carried out against monoclonal antibody SAF84 (an anti-PrP antibody with known binding to the epitope YRPVDQY). 48 phage clones were analysed from the output phage and 35 gave ELISA signals when binding to the immobilised SAF84 antibody (light grey bars, **a**) that were at least two-fold of that produced against a control monoclonal antibody (anti-PrP antibody SAF70, dark grey bars, **a**). Thirteen phages showing binding in the ELISA were sequenced and eight of these produced a consensus motif RPxxQY that matched the known PrP epitope (**b**). q, amber stop codon TAG

A single round of panning of the pVIII peptide library against the anti-PrP monoclonal antibody SAF84 was carried out. Resulting single bacterial colonies were picked and phage produced from them. ELISA screening of 48 of the phage demonstrated that 35 gave positive signals (Fig. 4a). Thirteen of these peptide genes were sequenced and eight coded for the consensus motif RPxxQY that matched the known PrP epitope YRPVDQY (Fig. 4b).

**Fig. 5:**
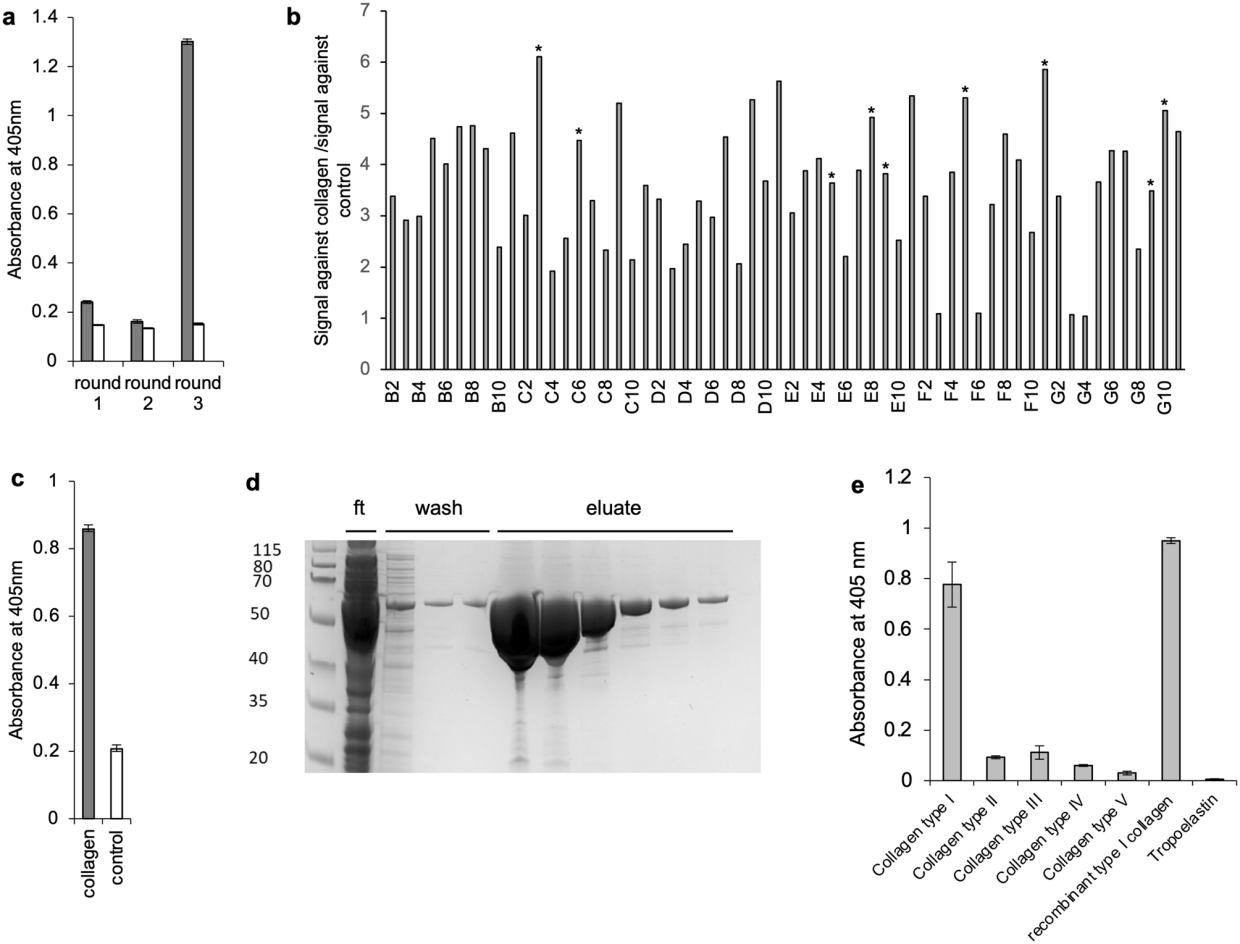
Panning for binders to collagen using the pSD pIII-VHH libraries. The three VHH libraries were combined and used to pan against recombinant collagen (20 μg/ml) immobilised on maxisorb plates. Following three rounds of panning, the polyclonal phage outputs were tested for binding to the target in an ELISA (**a**). Enrichment was observed with enhanced binding at round 3 to the target (grey bars) compared to earlier rounds and also when compared to a control protein (elastin, 20 μg/ml, white bars). Using the same assay, single colonies were picked from round 3, and 41 out of 60 displayed binding by ELISA that was at least three-fold higher than against the control protein (**b**, average of 2 repeat assays). Sanger sequencing of 9 clones (*) revealed the same CDR3 and binding was confirmed in a repeat ELISA (**c**). The VHH CDR3 region was cloned into the same scaffold VHH within the c5x expression vector to produce MBP–VHH fusion protein in the cytoplasm of *E. coli*. Affinity purification of the fusion on amylose resin demonstrated the one-step purification of VHH fusion (**d**). Column flow through (ft), three sequential wash steps and 6 sequential eluate aliquots are shown. The MBP–VHH fusion was then tested in ELISA (**e**) to confirm the specificity of the antibody (12.7 μg/ml) to 10 μg/ml collagen (types I to V and recombinant type I, as indicated) or against a control protein (tropoelastin at 10 μg/ml). ELISA data are displayed as average values for triplicates with standard deviations

The VHH libraries were pooled and panned over 3 rounds against immobilised recombinant human collagen. Enrichment for binding was demonstrated at round 3 by ELISA of the polyclonal phage (Fig. 5a). Output phage numbers were ~ 1 × 10^5^ for rounds 1 and 2 and ~ 1 × 10^7^ for round 3. Sixty individual clones were picked from round 3 and tested in ELISA, 41 of these gave signals at least three-fold higher than against the control protein (Fig. 5b). Sequencing of nine of these clones (*) showed that all had the same CDR3 sequence, ITAWGAPSQRDL. A single clone was produced as phage and reanalysed in ELISA, again demonstrating clear binding above the background (Fig. 5c). Using whole-plasmid PCR, this anti-collagen VHH CDR3 was cloned into the expression vector c5x containing the scaffold VHH in order to produce anti-collagen MBP–VHH fusion. Expression followed by purification of the whole cell extracts on amylose resin produced pure fusion protein at 42 mg/l of culture (Fig. 5d). The soluble VHH fusion was then confirmed as being specific for collagen type I and did not bind other collagen types (II to V) or the control protein tropoelastin (Fig. 5e). Within the ELISA the MBP–VHH could detect 62.5 ng of antigen (Fig. SI2).

## Discussion

The current study describes a method to rapidly produce phage display libraries using a one-step whole-plasmid PCR method to seamlessly introduce a region of high diversity. This was demonstrated for a 16mer peptide library displayed near the N-terminus of the pVIII protein and also for three VHH libraries where diversity was introduced within the CDR3 region. The method utilises a type IIS restriction enzyme cleavage site for seamless cloning of the insert and so the insertion site is completely adaptable for insertion at any site and within any ligand scaffold. The library diversity was reproducibly ~ 1 × 10^9^ from 10 transformations using ~ 1 μg of ligated DNA. This is a relatively simple method that produces diversity comparable to other established methods that often rely on multiple PCR steps and multiple restriction enzyme cleavage steps of both inserts and vectors before ligation, and the availability of multiple cloning sites in the vector [10, 11]. For antibody diversity, only a single VHH CDR was mutated by this method but iterative mutagenesis of other CDRs could be achieved by the same method. Alternatively, binders could be obtained from a CDR3 mutated library and affinity matured, if required, by the introduction of further diversity into CDR1 and/or CDR2 using the same method and the lead candidate(s) as the template.

The libraries were characterised by NGS analysis which described the extent of bias for peptide sequences within the region of diversity after propagation in bacteria but before display on phage. All libraries contained overrepresented sequences, as has previously been reported for both peptide and antibody libraries [12, 34, 35]. Here, overrepresented peptides seen 3 or more times made up between 3.4 and 5.3% of the sequences in the libraries and 82 to 86% of all sequences were present as single copies. Other pVIII peptide libraries have been analysed using NGS analysis after propagation in bacteria and display on phage. Two relatively low-diversity libraries using the same pC89 phagemid vector and insert site as the libraries described here, had 3% or 73% of clones with no insert and where insert was present, 94 or 95% of peptide sequences were present as single copies, respectively. Those peptides with a copy number of 3 or more represented 0.3% of the sequences [26, 31]. Ryvkin and co-workers produced several pVIII peptide libraries within a phage vector and the libraries contained from between 72% to just under 100% of sequences present as single copies [10]. Most reports of the NGS analysis of peptide libraries have used the commercial PhD-7 library [8], a pIII display library that uses a phage vector. Matochko and co-workers [36] demonstrated that overrepresented peptides seen more than 3 times made up 8% of the insert sequences in the library. The reported percentage of single copy number clones varied between studies for this library, which may reflect sequencing depth and/or variations in the preparation of the library; 58% [37], 72% [36] and 79% [10] of sequences have been reported as being present as single copies. A peptide library on pIII has recently been made using a similar whole-plasmid PCR construction to the one described here [12]; the deep sequence analysis of that library showed 93% of the inserts of the correct size insert, with nearly 98% of them being distinct sequences. The majority of the aberrant sequences were the PCR template. Here, the frequency of template clones was just 0.02–0.03% due to the effective use of the enzyme DpnI before library transformation. Ravn and co-workers [38] describe the production of synthetic and semi-synthetic single-chain Fv libraries and report that these had single copy number sequences for heavy chain CDR3 peptides between 80 and 99% of the sequences. Any bias at the clonal level is likely to be due to growth advantages of certain sequences over others when libraries are propagated in bacteria [36]. In the present study, libraries were sequenced directly after library production and growth of bacteria in the presence of glucose suppression of expression of the ligand gene-fusion and as such represent the basal level of sequence bias present in such libraries. It is possible this bias may be influenced by low-level ‘leaky’ expression of the ligand gene fusions and their relative toxicity but it is not influenced by the efficiency of packaging of the fusion protein in phage. Overall, the libraries contained bias similar to other libraries described in the literature. As the latter were sequenced after further propagation in bacteria and display on phage it may indicate the sequence bias is introduced very early on in library production and likely directly after transformation and propagation of the libraries in bacteria, even with low basal levels of expression from the ligand gene.

The NGS data were also used to analyse bias in terms of overall and positional amino acid usage. For overall amino acid usage, in the gene VIII peptide library Pro and Thr were the most overrepresented, and Val and Gly were the most underrepresented. This is consistent with other peptide libraries reported in the literature, where Pro is often the most overrepresented residue probably due to phage display favouring peptides with beta turn structures above alpha helices and beta sheets [39]. Pro was seen to be the most overrepresented residue across multiple libraries with both pVIII and pIII display formats [10, 31, 37, 39]. A pVIII phage library reported by Ryvkin et al. [10] did not have Pro as the most overrepresented, indicating this trait is not universal. Also, the library reported here has not been through any selection due to display on phage and so this cannot be the reason for this amino acid bias. Thr residues have also been reported to be highly overrepresented in multiple pIII display libraries [10, 37, 39]. The most often reported underrepresented residue is Cys probably due to single Cys residues being selected against as they can form aberrant disulphide bridges that may interfere with effective phage assembly and secretion [40]. Again, this is seen across multiple libraries and display formats [10, 31, 37, 39] with underrepresentation by − 2 to − 3%. Here, Cys was underrepresented by only − 1% in the pVIII peptide library which may reflect that the library had not yet been displayed on phage. Other libraries have also reported Val and Gly being underrepresent by − 1 to − 2% [31, 37] along with various other amino acids. Overall, the amino acid bias in the pC89 pVIII-16mer library is similar to other phage display peptide libraries and reflects the bias before display on phage.

In terms of the positional bias for amino acids in the pVIII peptide library, this was relatively consistent across all 16 positions and was relatively low with no bias greater than 1.1% of the theoretical distribution. Positional bias for other peptide libraries has been reported to be highest in the first 3 residues after the peptidase cleavage site due to bias in enzyme recognition [39]. However, the pC89 pVIII-16mer library has wild-type pVIII residues AEGEF at positions + 1 to + 5 after the cleavage position and so this region of possible bias is unlikely to be seen even after phage display. Other bias has been noted at position + 6, + 12 (Pro overrepresentation, [37, 39]) which is consistent with the present study in the + 6 position but again, for the library in the current study, this is not due to selective pressure during phage assembly and display.

Amino acid usage bias was not as pronounced for the pIII-VHH libraries; Asn was the most overrepresented amino acid. This residue has not been reported to be one of the most overrepresented residues in any of the previously reported peptide libraries [10, 29, 37, 39]. The most underrepresented residue differed between VHH libraries. The VHH libraries displaying 16mer and 21mer peptides had very similar patterns of amino acid positional distribution. The pattern of positional amino acid bias was distinct for the VHH 12mer library yet all three VHH libraries were cloned using the same methodology varying only in the length of the region of diversity. It seems likely that this variation is due to differences in the quality of the primers used in library construction. It is also of note that the patterns of positional amino acid bias and overall amino acid residue bias were distinct between the VHH libraries and peptide library which may reflect the different display formats exerting distinct selection criteria during bacterial propagation and/or variations in the efficacy or quality of the primers used in library construction. Codon bias will also be introduced into libraries due to different coupling efficiencies of the 4 bases when using machine mixed bases during oligonucleotide synthesis (as was the case here).

Having demonstrated that the libraries have high diversity and limited bias in terms of overrepresented peptides and amino acid usage, they were tested in terms of yielding ligands with specific binding. The peptide library was used to successfully epitope map a monoclonal antibody where the sequencing of thirteen binders revealed eight clones with a consensus motif that matched the known epitope. The panning of the VHH libraries against recombinant human collagen type I (Sigma) over 3 rounds produced a single dominant positive binder. The antibody CDR sequence could be cloned directly into an expression vector containing the scaffold VHH by whole-plasmid PCR. The recombinant MBP–VHH clone was produced at high levels in bacterial expression culture and retained its specific binding ability. This could represent an effective recombinant antibody synthesis strategy to allow direct cloning from NGS datasets describing the enrichment of phage during panning experiments, the so-called next-generation phage display. This is an alternative approach to using antibody libraries with multiple regions of heterogeneity, requiring the rescue of a particular antibody sequence (identified from NGS data) from a sub-library of enriched phage clones, which is a challenging and often inefficient process [40, 41].

Overall, the use of whole-plasmid PCR with type IIS restriction enzymes to facilitate seamless cloning of peptide diversity into phage display systems allows a rapid and relatively simple library construction method. Here, it was used to produce 4 libraries of more than a billion ligands with relatively low bias, and the libraries could be used to yield phage clones with specific binding characteristics.

## Supplementary Information

Below is the link to the electronic supplementary material.

Supplementary file1 (DOCX 922 kb)
