# Supplementary material for: A Simple Whole-Plasmid PCR Method to Construct High-Diversity Synthetic Phage Display Libraries
Source: Mol Biotechnol. 2022 Feb 2;64(7):791–803. doi: 10.1007/s12033-021-00442-4 (PMC9217769; doi:10.1007/s12033-021-00442-4)
Supplement: Supplementary file 1 — Supplementary file1 (DOCX 922 kb) [file 12033_2021_442_MOESM1_ESM.docx]

**Table S1** Probabilities of seeing a certain copy number of a sequence in each library NGS dataset

|  | PC89 pVIII-16mer^1^ | pSD3 pIII-VHH-12mer | pSD3 pIII-VHH-16mer | pSD3 pIII-VHH-21mer |
| --- | --- | --- | --- | --- |
| Poisson probability^2^ 1 | .977 | .999 | .999 | .998 |
| Poisson probability 2 | 9x10^-5^ | 2x10^-4^ | 4x10^-4^ | 1x10^-4^ |
| Poisson probability 3 | 6x10^-9^ | 3x10^-8^ | 1x10^-7^ | 1x10^-8^ |

^1^ The probability of seeing a sequence of 1 or more copy number divided by the probability of a sequence being seen in this copy number

^2^ number refers to the number of times seen in the library sequence data

**Table S2** The most overrepresented 25 sequences from each library

| PC89 pVIII-16mer |  |  | pSD3 pIII-VHH-12mer |  |  | pSD3 pIII-VHH-16mer |  |  | pSD3 pIII-VHH-21mer |  |  |
| --- | --- | --- | --- | --- | --- | --- | --- | --- | --- | --- | --- |
| Sequence^1^ | Copy number | % of sequences^2^ | Sequence^1^ | Copy number | % of sequences^2^ | Sequence^1^ | Copy number | % of sequences^2^ | Sequence^1^ | Copy number | % of sequences^2^ |
| DCSS | 3582 | 0.386 | VRGYFMRLPSSHNFRY | 212 | 0.024 | VRGYFMRLPSSHNFRY | 166 | 0.020 | VRGYFMRLPSSHNFRY | 143 | 0.027 |
| q | 2602 | 0.280 | VSSMqPIGRVII | 19 | 0.002 | KQKFAGNGSTKNSKNM | 19 | 0.002 | RGWTPRMHMELRDGPSLKRHQ | 34 | 0.006 |
| SS | 603 | 0.065 | DQAISSGVQEMA | 18 | 0.002 | RSSVMMAGDNAQFRAq | 18 | 0.002 | KMWQVEqKLRSHIHLLRDRDE | 26 | 0.005 |
| TDCSS | 470 | 0.051 | KVDMSRNWPSRL | 17 | 0.002 | SHIYACRVTAKDHVPA | 16 | 0.002 | FDNKAKLCDFQYAVKGAALQY | 25 | 0.005 |
| KAKNSACAAQNTSPFN | 397 | 0.043 | SqDTRCQGCNQT | 16 | 0.002 | GqRSS | 16 | 0.002 | ETDISWTMGYHTNRARRAWPN | 20 | 0.004 |
| DVNTTHATSLGTSRKR | 356 | 0.038 | RVWSAQDLqDLA | 16 | 0.002 | ESTEYNLWLMNYGYSN | 16 | 0.002 | LSVELKFIDCAALqRqYSDLS | 19 | 0.004 |
| PHSIRIVATTQTTAWM | 347 | 0.037 | NYRLKYCLRNGR | 15 | 0.002 | PRTNFNNKNNqVVNCH | 15 | 0.002 | RKPDTqTKHKYCIRTQPKFqR | 18 | 0.003 |
| ASDRHYKKHMVLEIN | 323 | 0.035 | YVVLGFSISSAC | 14 | 0.002 | TTVTSAQTNSGQCHIS | 14 | 0.002 | SVRSKMSLNITVHANGCSSHR | 17 | 0.003 |
| ICLR | 301 | 0.032 | VLEVHNNITQCR | 14 | 0.002 | QPVLYQVLNQSGRFTD | 14 | 0.002 | QqSqLESSDRYIRHRLREGIq | 17 | 0.003 |
| SIWAKTSYITPKLRPS | 274 | 0.030 | SCSNASNVVMAS | 14 | 0.002 | PTLLTLICKRTNIEPW | 14 | 0.002 | VADDGRMRGNqVNARTMVAYR | 16 | 0.003 |
| HANMSRDASPPCDSNP | 271 | 0.029 | PANVSNLLSTRV | 14 | 0.002 | NTTNWTHLRAQDDVQT | 14 | 0.002 | NRDMSGHWHGGWNLVISATGM | 16 | 0.003 |
| PDCSS | 265 | 0.029 | NKVLPNAHNDSC | 14 | 0.002 | HEVKYNLLLRGNPDEL | 14 | 0.002 | LGHSLEYDDRAHASDTPGRNK | 16 | 0.003 |
| KPLHQTTARQQPNARS | 249 | 0.027 | GDTLCLPGMCCI | 14 | 0.002 | ARPIYHSLPTRSDSLP | 14 | 0.002 | SGSVTCSVKDqNWqRMSGEWM | 15 | 0.003 |
| RGYHPRRYSqTDGNRT | 243 | 0.026 | YTVRARVLMRYM | 13 | 0.001 | THITSqGVYLSIqVAI | 13 | 0.002 | PNTDPGRSDEHARYTDRTMHS | 15 | 0.003 |
| qDCSS | 239 | 0.026 | VqFTNERLFNNY | 13 | 0.001 | qTAVQNLRDDMqMLYL | 13 | 0.002 | LVGWQLGNRCNKSIAHAVGFN | 15 | 0.003 |
| TCAKAKHRESEPRKER | 236 | 0.025 | VILHHSGIWVSL | 13 | 0.001 | PPNLRTGMIVFAATSR | 13 | 0.002 | VASVGYINPASILSHLSVDKT | 14 | 0.003 |
| RDCSS | 222 | 0.024 | TRDDSKIVRGKA | 13 | 0.001 | MNTEKSYPPDCWHQSA | 13 | 0.002 | RTRMVPIKQQYKLqGYTDCHR | 14 | 0.003 |
| QDCSS | 219 | 0.024 | RRECCIqLGSSL | 13 | 0.001 | KCAKPRGAYWARIDTR | 13 | 0.002 | NVAHDKLGqNSTEMYqSAKWI | 14 | 0.003 |
| SDCSS | 212 | 0.023 | QMGYSRWQGAYV | 13 | 0.001 | GKDILYTCTVLPCNHG | 13 | 0.002 | NRPIPRTGCRLPqADRSWTAI | 14 | 0.003 |
| KDCSS | 211 | 0.023 | PTTDSRGqLAIK | 13 | 0.001 | YATKSSQTQRTIASQT | 12 | 0.001 | MLPIMVSMAREAVKGQNNSLT | 14 | 0.003 |
| KTRAHFIDSHIPHTSI | 203 | 0.022 | MGCLERLFQESS | 13 | 0.001 | IYGDCYDLVHSRIKTR | 12 | 0.001 | MAKWYSTNPENKLHTRVDGIR | 14 | 0.003 |
| PTTPMPTWLSKAKSGR | 200 | 0.022 | LQDRPWNFYLFP | 13 | 0.001 | HRETRLSLCNEKQLDV | 12 | 0.001 | KMRKLDGSRYYTCLLNMRRSG | 14 | 0.003 |
| P | 198 | 0.021 | KRSTRMQAGPRA | 13 | 0.001 | HMSKLNLRFAPRIEPR | 12 | 0.001 | IVTKMSFPDSTKRIGSMTqQP | 14 | 0.003 |

^1^ q is amber stop codon; ^2^sequences that contained an in frame insert with flanking regions

**Figure S1** The VHH-pIII display phagemid. The VHH CAbBII10 scaffold sequence is shown in frame with C-terminal affinity tags (a). The site of VHH cloning within the phagemid pSD3 between the pelB leader sequence and the truncated pIII gene is also shown with an amber stop codon between the VHH-affinity tags and the gIII gene (b and c). For VHH, FW - framework region, CDR - complementarity determining region. Figure created using SnapGene viewer version 4.3.10 (snapgene.com).

**Figure S2** The limit of detection for the MBP-VHH fusion binding to collagen type I by ELISA was determined. The antibody (50 μg/ml) binding to a dilution series of collagen immobilised on the ELISA plate (as indicated in ng per well) was determined. The signal against a no antigen PBS control (0.114) was subtracted from all values before plotting. A control of tropoelastin (500 ng added to each well) was used as a further control and the MBP-VHH could consistently detect 62.5 ng of collagen above background (control antigen) in this assay. The assay was carried out twice and gave equivalent results. ELISA data is displayed as average values for duplicates with standard deviations.
